# Negotiating with digital self-monitoring: A qualitative study on how patients with multiple sclerosis use and experience digital self-monitoring within a scientific study

**DOI:** 10.1177/13634593231175321

**Published:** 2023-05-17

**Authors:** Karine Wendrich, Lotte Krabbenborg

**Affiliations:** Radboud University Nijmegen, The Netherlands

**Keywords:** digital self-monitoring, lifeworld, multiple sclerosis, patient participation, technology assessment

## Abstract

Research shows that patients can have values and use practices that are different from those envisioned by technology developers. Using sociomaterialism as an analytical lens, we show how patients negotiated with digital self-monitoring in the context of a scientific study. Our paper draws on interviews with 26 patients with the chronic neurological disease multiple sclerosis (MS) who were invited to use an activity tracker and a self-monitoring app for a period of 12 months as part of their everyday life. Our study aims to fill a gap: relatively little is known about how digital self-monitoring becomes materialized in the everyday lives of patients with chronic diseases. We show that patients engaged in digital self-monitoring because they are eager to participate in research to contribute knowledge that will benefit the larger community of patients rather than to improve their personal self-management. Although respondents adhered to digital self-monitoring during the study, it is not self-evident that they would do so for private self-monitoring purposes. It became clear that respondents did not necessarily perceive digital self-monitoring as useful for their self-management practices due to their established knowledge and routines. Moreover, respondents referred to the inconvenience of having to perform self-monitoring tasks and the emotional burden of being reminded of the MS because of the digital self-monitoring. We conclude by indicating what could be considered when designing scientific studies, including the suitability of conventional study designs for evaluating technologies used daily by patients and the challenge of integrating patients’ experiential knowledge into scientific practices.

## Introduction

Digital self-monitoring refers to the use of mobile health technologies, such as smartphones and activity trackers, for collecting and measuring personal health data on bodily functions and everyday activities (Lupton, 2013, 2017; Sharon, 2017). Digital self-monitoring is surrounded by the assumption, as articulated by technology developers, health innovators and policy makers, among others, that this innovation will improve healthcare and patients’ lives (Sharon, 2017). Promises include the technology enhancing the efficiency and quality of patient care and facilitating the self-management of chronic diseases. The expectation is that these technologies will support patients to gain knowledge about their bodies and that this will make them feel more in control of their health and more engaged in the management of their disease (Lupton, 2013; Sharon, 2017).

However, research in the fields of, among others, science and technology studies (STS) and medical humanities has already demonstrated that users, including patients, can have ideas, values and use practices that can diverge from those envisioned by the developers of a technology (Oudshoorn, 2011; Pols, 2012). Although technologies contain “scripts,” that is, explicit and implicit directions for how and by whom a technology should be used, this does not guarantee that these directions will actually be carried out (Akrich, 1992). Users do not passively execute what is being prescribed to them, but can actively change, resist and challenge a technology’s instructions. Regarding digital self-monitoring technologies specifically, it has been found that they can be rejected by potential users, used only temporarily or intermittently or used in a different way to that intended by the developers (Birkhoff and Smeltzer, 2017; Weiner et al., 2017). For instance, Lazar et al. (2015) found that participants in their study abandoned digital self-monitoring devices because they did not fit participants’ conceptions of themselves or because the self-monitoring data were not perceived as useful.

It turns out to be difficult to predict how a technology such as digital self-monitoring will behave “in the wild,” that is, when introduced in people’s daily lives, as technologies will ultimately get their meaning and function through their interactions with users (Pols, 2012; Pols and Willems, 2011). In fact, in their study on a smartphone app for diabetes, Ledderer et al. (2019) argue, inspired by the work of Lupton (2016) and Marres (2012), that “as such, it is not so much about how or to what extent patients use apps to participate in their health, but rather how apps and the contexts in which apps are produced and used make certain forms of participation possible” (p. 2). It is this sociomaterial perspective (Lupton, 2016), that is, the recognition that digital self-monitoring technologies can be considered material actors that interact with human actors and as such enable and constrain certain forms of behavior and activities, that we employ in our analysis.

Until the time of writing, several empirical studies have been conducted on the use of digital self-monitoring technologies in people’s everyday lives. Whereas some researchers have investigated patients with chronic diseases (e.g. Danesi et al., 2020, 2021), most empirical research thus far has focused on healthy people who have started using digital self-monitoring based on their own decisions and with the aim of improving their lifestyle (see, for instance, Didžiokaitė et al., 2018; Lupton, 2019a; Presset et al., 2021; Sharon, 2017). At the time of writing, relatively little is known about how digital self-monitoring technologies come to matter in the everyday lives of patients with chronic diseases (Maslen and Lupton, 2020; Weiner et al., 2017). People with a chronic disease assess digital self-monitoring differently from healthy people and might have different motivations for self-monitoring their health. As Meidert and Scheermesser (2021) found, healthy people engage in digital self-monitoring primarily out of fun and curiosity, whereas people with a chronic disease mainly use it to manage their disease. Therefore, as suggested by other scholars (Sharon, 2017; Weiner et al., 2017), there should be a widening of the empirical focus to consider a more diverse range of user experiences to do justice to the complexity and variety of self-monitoring use and to gain a better understanding of the dynamics of (non-)use. Such an inquiry will inform and enrich critical discussions in the literature on the real-life implications of digital self-monitoring technologies, in particular for patients with a chronic disease.

In this paper we contribute to filling this gap by focusing on patients’ engagement with digital self-monitoring in the context of a scientific study. At the time of writing, many digital self-monitoring technologies for chronic diseases are not widely used in society but are still at the research phase. To assess medical innovations before their implementation in clinical practices, it is common for researchers to engage in evaluation studies that follow standardized and controlled study designs, like randomized controlled trials (RCTs) (Naaldenberg and Aarts, 2020; Pols, 2012). During these evaluation studies, patients are invited to use a medical innovation and usually have to adhere to standardized study protocols (Pols, 2012). These study protocols typically follow a one-size-fits-all logic in which patients’ use of the technology, such as the frequency of use and the time of day when the tasks are performed, is prescribed by the researchers. These protocols therefore both enable and constrain the actions and behaviors of the patients.

We aimed to gain insights into what patients actually do with digital self-monitoring technologies during a scientific study, such as (dis-)obeying instructions or challenging or questioning what is being prescribed to them by the study protocol. Inquiring into patients’ use practices and experiences during a study is valuable as it allows evidence to be gathered about patients’ willingness to use digital self-monitoring at an early stage of technology development, including facilitators that aid or encourage use and barriers that prevent use. In our study we considered patients with the chronic neurological disease multiple sclerosis (MS) who engaged in digital self-monitoring for 12 months in the context of a scientific study called the “APPS MS study” (Lam et al., 2022). The APPS MS study was initiated by an academic medical center in the Netherlands in close collaboration with two companies developing self-monitoring apps, and its aim was to evaluate the validity and reliability of digital self-monitoring technologies for monitoring MS. Participants who took part in this study were invited to use a Fitbit Charge 4 activity tracker and an MS-specific smartphone app as part of their everyday life while adhering to the study protocol set by the scientists involved in the study (see “Research setting” in the Methods section). In this paper we focus on how patients engaged with these two digital self-monitoring technologies during the APPS MS study, taking the sociomaterial notion of “fitting” (Pols, 2012), as further described in the next section, as starting point.

## Fitting technologies into patients’ everyday lives

Sociological scholars from STS and medical humanities, as well as those from other academic subjects, have devoted a significant amount of time to studying how patients interact with new technologies in their everyday lives (Hyysalo et al., 2016; Oudshoorn and Pinch, 2003). Many scholars in this field have described the discrepancies between the intended use of technologies as envisioned by technology developers and how patients adapt a technology to make it useful in the context of their everyday life (Oudshoorn and Pinch, 2003). According to sociomaterial theories, when a new technology is introduced in the daily practices of users, it interferes with their existing everyday reality, such as established knowledge, infrastructures and daily routines (Danesi et al., 2020; Danholt and Langstrup, 2012). The technology needs to be transformed from an unfamiliar thing into a familiar object that is incorporated within “the physical and social environment of the patient” (Danesi et al., 2020: 2016). During this process, new sociotechnical configurations emerge in which patients adapt the technology in order to match their established practices, but patients’ practices can also be adapted to better align with the technology (Danesi et al., 2020).

When the process of building a relationship between the patient and the technology results in a situation in which the technology is useful and meaningful for patients’ needs, goals, activities and routines, this is called a “fit” (Pols, 2012, 2017). People are more likely to adhere to technologies in the long term when there is a “fit” between the user and the technology. For instance, Presset et al. (2021), who studied the use of an activity tracker provided by a health insurance company, found a strong alignment between the material and the social, that is, between the technical features of the technology and the social values of the users, resulting in an overall acceptance of the technology. But there can be a lack of fit or a bad fit between humans and technologies, meaning that there are collisions or tensions between material and social actors, with technologies interfering with rather than facilitating people’s everyday life (Pols, 2012, 2017). This also often seems to be the case for digital health technologies such as digital self-monitoring, which end up not being used by patients because the technology is not in line with their needs, desires and daily practices (Ancker et al., 2015; Dam Nielsen, 2015). Whereas technology developers might assume that patients are enthusiastic about collecting their personal health data through technology and engaging in self-management of their health, the reality is that patients might not share the underlying values of the technology, such as adopting a proactive attitude and engaging in routine monitoring of their health (Ancker et al., 2015; Lupton, 2013).

Although this distinction between fitting and not fitting, or not fitting well, with people’s daily lives and values suggests that only a binary outcome is possible—that people either embrace or reject a technology—it should be noted that the reality is more dynamic and ambivalent than this. Often people shift between using and not using a technology over time, depending on how their circumstances, needs and values evolve (Weiner and Will, 2016). Moreover, people can be selective in their engagement with a technology, for instance by only using particular functions of the technology or only using the technology in certain circumstances. Oudshoorn (2008) has coined the term “selective use” for this phenomenon. By acknowledging the diverse and ambivalent ways in which people interact with technologies in their day-to-day life, we aim to analyze how the MS patients negotiated with the digital self-monitoring technologies during the APPS MS study and how well these technologies fitted patients’ lifeworld.

The phenomenological concept lifeworld refers to the everyday experiences, activities and circumstances that construct the subjective reality of an individual person (Kraus, 2015). We view this lifeworld as the frame of reference through with patients assess their engagement with new technologies The lifeworld of MS patients is characterized by uncertainty and a lack of control, as the disease is highly unpredictable and varies significantly between patients (Ayobi et al., 2017; Cowan et al., 2020; Dennison et al., 2016). In the majority of cases, MS is characterized by alternating periods of relapse, during which symptoms deteriorate, and periods of recovery. The symptoms that MS patients might experience include mental and physical fatigue and problems with cognition, mobility, hand function and vision (Knaster et al., 2011; Simblett et al., 2019). Another central characteristic of the lifeworld of MS patients concerns loss, such as losing independence, employment, mobility or other functional abilities (Cowan et al., 2020; Preston et al., 2014; Tabuteau-Harrison et al., 2016).

MS also results in a changed relationship to one’s body and self (Preston et al., 2014). After the diagnosis, MS patients need to find a way to live with their MS and their “new” body, which feels and behaves differently from the way it did before the onset of symptoms (Cowan et al., 2020; Dennison et al., 2016). MS patients are known to engage in various forms of self-management, which refers to the use of interventions, education or skills by patients, for example, seeking information online or adopting lifestyle habits, to (learn how to) manage their illness, such as the effects of the disease on their daily lives (Ayobi et al., 2017; Dennison et al., 2011; Knaster et al., 2011). As we will show, these existing self-management practices form the background against which patients negotiated with the digital self-monitoring technologies.

## Methods

### Research setting

During the APPS MS study, patients used a Fitbit Charge 4 activity tracker and a prototype of a smartphone app for self-monitoring MS for 12 months. The aim of this scientific study was to investigate the correlation between the digital self-monitoring data and traditional clinical assessments for MS in order to assess the reliability and validity of these digital tools for monitoring the health of MS patients.

The Fitbit Charge 4 is a smartwatch that collects data on patients’ physical activity, heart rate and sleeping patterns. Patients had to wear this smartwatch for the entire duration of the APPS MS study. They were also instructed to use the MS self-monitoring app to answer eight daily questions on mental wellbeing (e.g. mood, energy and stress) and to perform five weekly tests, namely three fatigue tests, one walking performance test and one cognitive performance test. Each of these five tests had to be executed on a fixed day of the week. The assumption is that when the same measurements are performed each week, comparisons can be made of patients’ health over time. The three common features of the activity tracker and the self-monitoring app are that they reduce health to quantifiable measures, do not take users’ context, that is, everyday activities such as work and caring responsibilities, into account and require people to be proactive and responsible (cf. Presset et al., 2021).

First, the activity tracker reduces health to steps, heart rate and sleep quality, and the smartphone app reduces health to fatigue, walking performance and cognitive functioning. There seems to be limited space for patients’ subjective, non-quantifiable experiences of health. Second, both technologies assume that people are willing and able to engage in digital self-monitoring tasks on a regular basis, and there is limited consideration of their everyday life context, such as their paid employment and doing household tasks, that might constrain patients’ ability or willingness to engage in digital self-monitoring. Third, both technologies make patients responsible for monitoring their health and acting in response to the results, which is in line with neoliberal ideologies that envision patients being in charge of their own health (Lupton, 2013; Sharon, 2017). It is these implicit and explicit assumptions that are inscribed in the technologies, and which encourage certain forms of use while discouraging other forms (Akrich, 1992), that we investigated further in the interviews.

### Data collection

Interview respondents were recruited via the medical researcher based at the academic medical center coordinating the APPS MS study. Interested potential study participants were invited to contact the first author by email. The first author was contacted by 34 patients, 26 of which were interviewed. Patients were included on a “first come, first served basis.” As data saturation occurred after 26 interviews, no additional respondents were included.

The 26 interviews were conducted in Dutch between January and March 2021 by the first author, a PhD researcher in science and technology studies who has prior experience in conducting interviews with MS patients. Due to the Covid-19 pandemic, 23 interviews took place via video call (Google Meet) and three interviews via telephone. Written informed consent was obtained before audiotaping the calls, as approved by the Research Ethics Committee of the Faculty of Science (REC19012). The interviews lasted from 58 to 96 minutes, with an average duration of 74 minutes. The respondents were interviewed between 1 and 19 months (6 months on average) after they had finished their participation in the scientific study. All of the interviews were audiotaped.

A semi-structured interview protocol was developed by the first and second authors; the latter is an associate professor in science and technology studies and is experienced in conducting interviews with patients about the possible effects of medical innovations on their everyday life. The interview guide discussed (1) patients’ motivation for taking part in the scientific study; (2) how patients integrated the digital self-monitoring technologies into their daily practices and what difficulties they experienced in this regard; (3) how patients acted on the digital self-monitoring data; and (4) their envisioned future use of digital self-monitoring. The same interview guide was used for all interviews, but the semi-structured set-up of our interview guide allowed for some flexibility, for instance to ask probing, open-ended questions on topics mentioned in previous interviews.

### Data analysis

A combination of categorizing and connecting strategies was used in the analysis of the interviews (Maxwell, 2012). First, the interviews were transcribed verbatim using Microsoft Word 2016. Ten interviews were transcribed by the first author and the other 16 were transcribed by two student assistants. Next, both authors familiarized themselves with the data by reading the transcripts a couple of times. The transcripts were then uploaded to the qualitative data analysis software ATLAS.ti 8 and subjected to a predominantly deductive analysis which was guided by a codebook (Braun and Clarke, 2012, 2022). The codebook was based on theory and the aforementioned assumptions embedded in the digital self-monitoring technologies and was complemented with inductive codes arising from the data. When the data were being organized, deductive and inductive codes were separated from one another in the codebook. After the first author had coded the interview transcripts, the first and second authors engaged in regular discussions with each other to identify patterns in and relationships between the different codes. This resulted in themes and subthemes being identified that contained the same idea or concept. Quotes translated from Dutch (by the first author) are used throughout the Results section to illustrate our findings.

## Results

Our interview sample included 17 females and nine males. The respondents’ ages varied between 28 and 64 years, with an average age of 49. At the time of the interview, the respondents had received their MS diagnosis 2 to 34 years ago (14 years ago on average). We will discuss how the interview respondents used and experienced the MS self-monitoring app and activity tracker during their participation in the APPS MS study. The respondents appeared to assess the digital self-monitoring technologies from the perspective of contributing to scientific research. They expressed altruistic motivations rather than individual health optimization as reason for engaging in digital self-monitoring during the scientific study. Moreover, the respondents did not talk about the activity tracker and the smartphone app as isolated artifacts but evaluated them from the perspective of their lifeworld. It became clear that digital self-monitoring does not necessarily fit respondents’ lifeworld and can in fact be constraining rather than enabling for patients’ day-to-day life.

### Contributing to scientific research

Developers of digital self-monitoring technologies assume that patients are motivated to engage with these technologies so that health data can be collected for personal purposes and in relation to their individual goals (Ancker et al., 2015). During the APPS MS study, the interview respondents mainly used the MS self-monitoring app and Fitbit activity tracker because they were motivated by altruistic reasons, that is, they wanted to contribute to scientific research on MS and help future generations rather than being motivated to use the technology for their own personal gain:

MS14: Well, probably I will not benefit from it any more, but I hope that it yields something for the generation after me. That they find something that benefits MS.

Patients’ use of digital self-monitoring during the study was shaped by this altruistic motivation, which was particularly the case for the self-monitoring app. Respondents were motivated to use the app as instructed and tried to adhere to the weekly tests and daily questions, as they wanted to provide the researchers with as much useful data as possible:

MS16: You feel responsible. So you continue with it. You think: I have accepted this adventure. It is larger than I am. It is not about me. It is about people. You commit to something, you want to finish something, then you also have to do that. [. . .] I have followed the prescriptions as much as possible. I thought: I participate in research. If I do not track those data well, then they will miss data. Then they do not have good data. Then I am not a good representant of the population.

Although respondents followed the instructions of the self-monitoring app during the study, it is not self-evident that they would also do so if they were not motivated by wanting to contribute to scientific research:

MS8: Because you understand the importance of the research, you are doing it. But if this was structural, then I am not going to do it any more at a certain moment. So I was aware of the fact that if this was not research, I would have been less loyal toward the app.

Our findings echo previous observations made by Presset et al. (2021), who acknowledged that people are more likely to adhere to digital self-monitoring technologies in the context of a scientific study, as they feel responsible for contributing to the study.

### Self-management needs and practices

Whereas the Fitbit activity tracker and the MS self-monitoring app were meaningful to the respondents in the context of contributing to scientific research, it turned out that using these technologies for private self-monitoring purposes (Lupton, 2014) did not necessarily fit patients’ lifeworld. Patients’ existing self-management needs and practices appeared to play an important role in this regard. Both the self-monitoring app and the activity tracker are aimed at facilitating self-management. The app does this by providing MS patients with knowledge about their health status, whereas the activity tracker stimulates users to pursue a healthy lifestyle. It became clear that many interview respondents did not self-evidently perceive these technologies as useful for their self-management practices due to their established knowledge and routines.

#### Experiential knowledge

Self-management of MS becomes easier over time, as patients gain experiential knowledge and become familiar with the particular symptoms that they experience (Dennison et al., 2011). Experiential knowledge is the knowledge that patients have based on their everyday life experience with a certain disease (Boenink et al., 2018; Pols, 2012). Patients’ experiential knowledge appeared to play an important role in respondents’ assessment of the MS self-monitoring app.

The majority of the interview respondents were of the opinion that they do not need the knowledge provided by this app, as they mostly rely on their experiential knowledge when self-managing their health. During the interviews it turned out that especially those who have had MS for many years believe that they know their body very well and listen to their body to get an indication of how they are doing and to guide their self-management practices:

MS21: I know how I am doing. My body has two important parts. If they are doing well, then I know I am doing worse. My feet. Those are my indicators. At the moment my feet start too tingle. If I have done too much. Have used too much energy from my body. If my feet start to tingle. Then I know: if I continue for too long, then I have the risk that I will go toward a relapse.

Because of their experiential knowledge, most respondents were hesitant about continuing their use of the app for private self-monitoring, that is, outside the context of a scientific study:

MS4: I have insight into how I am doing at the moment. I have no reason to believe that that will change on short notice. And then I just do not find it necessary to do a test every day. Then I am too much occupied with it, whereas now there is no reason for it.

Several respondents believed that digital self-monitoring might be a better fit for MS patients who have just received the diagnosis, as they still need to gain experiential knowledge about their body and the disease. Despite their hesitance about using the app for private self-monitoring, many of the respondents said they could imagine using a self-monitoring app if the data were to be followed up by healthcare providers as part of the medical treatment:

MS3: When the neurologist really wants to do something with it. Yes, then I would be okay with that, that is not a problem at all. I think that is the most important for this kind of app. It needs to serve a purpose. If it does not serve a purpose, then it does not have so much added value.

This shows that whether a technology fits the patient’s lifeworld depends on the context in which the technology is put to use. Whereas the majority of the respondents seemed to resist the use of the self-monitoring app purely for private self-monitoring purposes, they appeared to agree with using the app for therapeutic purposes.

### Pursuing a healthy lifestyle

A healthy lifestyle turned out to be a key component of respondents’ self-management needs and practices. Indeed, adopting lifestyle habits, such as regular physical exercise, is a common self-management practice among MS patients (Ayobi et al., 2017; Knaster et al., 2011; Simblett et al., 2019). Multiple respondents explained that for them a healthy lifestyle, and physical activity in particular, is important for dealing with their MS. They try to keep their body as healthy as possible by maintaining a healthy lifestyle, and this gives them a sense of control, as they see a healthy lifestyle as a way to reduce the impact of the disease on their everyday life:

MS8: I am really searching for the limits of what I am able to do with my MS. I am searching a lot for the balance between physical fitness and accepting that my body cannot do certain things. [. . .] Of course I understand that MS cannot be managed for a lot of things. I am actually hoping that the part that can be managed, I hope that I can manage it as long and as much as possible.

Activity trackers can motivate people to pursue a healthy lifestyle, as their design stimulates users to increase their physical activity. Indeed, for some of the interview respondents, digital self-monitoring played a central role in managing their lifestyle. At the time of the interviews, the majority of the respondents were still using an activity tracker either because they were already using a smartwatch prior to their participation in the APPS MS study or because they opted to keep the Fitbit activity tracker after they had participated in the study. Even though a substantial proportion of the respondents were using an activity tracker, there were considerable differences between respondents regarding how they interacted with the activity tracker and how they acted on the data, ranging from respondents who were just monitoring their sleep sporadically out of fun and curiosity and did not act on the resulting data to respondents who kept track of their physical activity, heart rate and sleep every day and used this information daily when making decisions about their everyday life.

MS15: With the Fitbit I looked every day, I think 100 times a day. I still do. It is just addictive. I wake up and the first thing that I do is look: how have I slept?

These findings indicate that multiple, heterogeneous use practices emerged as patients negotiated the activity tracker. The interview respondents adapted how they used the activity tracker and turned it into a practice that was useful and meaningful for them (Pols, 2012, 2017). It became clear that whether or not the activity tracker fitted respondents’ lifeworld depended on their self-management needs. Several respondents found that the step counting motivated them to maintain or increase their physical activity, which was in line with their desire to pursue a healthy lifestyle as part of their self-management practices:

MS4: I look very regularly whether I have achieved those 10,000 steps. Not that it needs to be 10,000 exactly, but for me it has become a guideline. That I am just trying to be and to stay physically active.

However, multiple respondents were not motivated by the activity tracker to engage in a healthy lifestyle, either because they did not have this self-management need or because they had established other practices and routines to fulfill this need, which made the activity tracker unnecessary for them (cf. Shin et al., 2019). For instance, three respondents explicitly mentioned that they had purchased a dog to keep them physically active:

MS23: I have a dog, specially purchased, and she will make me walk. You know, I am like: I do not need to know exactly how many steps.

Our findings confirm observations made by Meidert and Scheermesser (2021) and Apolinário-Hagen et al. (2018), who found that patients with a chronic disease, such as MS, are mainly motivated to engage in self-monitoring for self-management purposes. For some of the respondents in our study, digital self-monitoring technologies such as activity trackers seem to fulfill a need, whereas for others these technologies are not meaningful as they have already found other ways to manage their disease.

### The burden of digital self-monitoring

The experienced burden of digital self-monitoring is another factor that appeared to influence how well these technologies fit patients’ lifeworld. Digital self-monitoring technologies are positioned by their developers as easy to use and requiring minimal effort (Danesi et al., 2020; Hortensius et al., 2012). However, the material design of these technologies implies that users need to engage in regular tasks, and they are confronted with their data, which does not necessarily fit with patients’ established practices and routines (cf. Ancker et al., 2015). Several of our interview respondents referred to the burden they experienced when using digital self-monitoring. This burden seemed to be twofold: the inconvenience of having to perform the self-monitoring tasks and the emotional burden of being reminded of the MS.

Multiple respondents mentioned the inconvenience of using the MS self-monitoring app, as this technology requires active user participation to adhere to the self-monitoring tasks. Despite being motivated to contribute to scientific research, several respondents experienced challenges in this respect. The assumption underlying the self-monitoring app is that MS patients have to perform the same tests each week so that their health can be monitored and compared over time, but some respondents became bored and annoyed by having to perform the same tasks over and over again:

MS12: In the beginning I liked it. At a certain moment it becomes monotonous and it is the same exercises every day and then the fun goes away. Every time the same exercises, there is no variation at all. Every week it is the same and at a certain moment I started to dislike it.

Furthermore, self-monitoring was perceived by some of the respondents as more than just a small effort. They had to do various activities throughout each day, such as performing tests and answering questions. Consequently, for several of the respondents, interacting with the self-monitoring app felt like an “obligation” rather than a pleasant activity:

MS25: It really felt, it was a real effort, what I had to do for it. Yes, of course it did not mean a lot, but still, you had to sit down for it for a couple of minutes.

It turned out that the design of the self-monitoring app did not fit the daily practices of some respondents, who struggled to a significant extent to make time for the app due to their other everyday tasks, such as their paid employment or childcare commitments. This made it difficult for them to integrate the app into their daily routines:

MS26: In the morning I was working and then I was resting and then I was busy with housework, cooking, that kind of stuff. I don’t know, then it was just not part of it [my routine].

Besides the inconvenience of routinely engaging in digital self-monitoring, self-monitoring data can also be emotionally burdensome for patients, evoking sensations like disappointment or pressure (Ancker et al., 2015; Ayobi et al., 2017). The design of the Fitbit activity tracker and MS self-monitoring app forced interview respondents to be reminded of their disease multiple times per day. For instance, the activity tracker would confront them with the fact that on a particular day they were unable to meet the required step count. Moreover, for the self-monitoring app, they had to perform tests and answer questions which were related to MS. It turned out that respondents did not necessarily want to be reminded of their MS, as they generally wanted to focus on the positive things in life, such as what they can still do rather than being frustrated or sad about what they cannot do any more:

MS19: Be happy with what you can still do. And don’t look back at what you cannot do any more. You just have to accept it. That is MS. You have to accept everything what happens.

In our interview sample the emotional burden of the digital self-monitoring seemed to depend on how the respondents were identifying with their MS. A substantial proportion of our respondents appeared to have accepted their MS. These respondents did not really seem to be negatively affected by the self-monitoring data:

MS9: I am not happy or depressed when I have had a good or a bad day. You just take it as a fact. That is the variability of MS. There is nothing you can do about it. [. . .] Everyone deals with it in his own way. Another person gets angry when he cannot take so many steps. Then I think: well, if not today, then tomorrow. But that is also my way of dealing with the MS. Grasp the day.

However, we also talked to interview respondents who were finding it more difficult to accept their MS and wanted to think about the disease as little as possible:

MS13: If I am not thinking about it, if I am not occupied with it too much, then it’s not there. Actually, I would rather not talk about it; actually I deny it.

The design of the digital self-monitoring technologies made these respondents have a negative confrontation with their MS. This especially appeared to be the case for some of the respondents who had received the diagnosis just 1 or 2 years before their participation in the APPS study and who were still in the process of accepting their MS. For these respondents the digital self-monitoring initially resulted in a lot of frustration and disappointment, as it made them aware that they were not able to function as a healthy person any more because of their MS. However, over time they learnt that it is better to accept their MS than to fight against it, which made the digital self-monitoring less emotionally burdensome for them:

MS17: For myself I have the goal to walk 10,000 steps each day. But if that’s not possible, then you also have to accept that it’s not possible. And you also have to learn that. In the beginning that was really difficult for me, if it was not possible. And eventually I came to the conclusion: well, 10,000 steps is also just a number, you do not have to achieve that. Well, and then you get a step further in the process that you also have to let go of things. Also the number 10,000.

Our findings challenge technology developers’ assumption that patients are willing and able to engage in digital self-monitoring tasks on a regular basis, as the self-monitoring data can have a negative emotional impact and the constant engagement with these technologies can result in boredom, annoyance and frustration (Ancker et al., 2015; Lupton, 2019b).

## Discussion

By taking a sociomaterial approach, which we have done by showing how MS patients negotiated a self-monitoring app and an activity tracker in the context of a scientific study, our findings show that patients questioned and challenged several assumptions underlying digital self-monitoring. During the scientific study patients had to use digital self-monitoring as part of their everyday life for a period of 12 months and had to follow a one-size-fits-all protocol in which the frequency of using the technologies, in this case on a daily basis, and the time of the day when the tasks had to be performed, were prescribed by the researchers.

The context of participating in a scientific study turned out to be conducive for how patients used and evaluated the digital self-monitoring technologies in their everyday life, especially the self-monitoring app. We found that patients engaged in digital self-monitoring because they are eager to participate in research to contribute knowledge that will benefit the larger community of patients rather than to improve their personal self-management practices (cf. Dam Nielsen and Langstrup, 2018). It became clear that patients might perceive private self-monitoring, that is, the use of digital self-monitoring for personal health optimization purposes (Lupton, 2014), as not fitting well with their existing knowledge, practices and routines. Similar to Ledderer et al. (2019), who demonstrated that a diabetes app did not fit well with patients’ daily lives, “as it presupposed ways of interacting with the app that were not well-aligned with their needs and preferred practices” (p. 10), we showed that the patients in our interview sample have established practices for managing their MS that might conflict with the material characteristics of the digital self-monitoring technologies. For instance, patients might resist being confronted with their MS and become annoyed or frustrated by having to adhere to self-monitoring tasks on a regular basis.

Whereas technology developers assume that digital self-monitoring facilitates patients in their self-management, our findings illustrate that MS patients already engage in several self-management practices, such as staying physically active and balancing their energy levels (Ayobi et al., 2017; Knaster et al., 2011). Patients with a chronic disease are known to develop skills and expertise that help them to accommodate their condition in their everyday life (Storni, 2014). We have shown that this experiential knowledge about living and dealing with MS seemed to reduce patients’ need to engage in digital self-monitoring as part of their self-management. This especially seemed to apply to patients who have been diagnosed with MS many years ago. Technology developers should therefore not assume that their norms and assumptions, which configure a certain type of patient and a specific perspective on the human body, in this case the proactive, digitally engaged patient whose bodily functioning is expressed in numbers and graphs, are shared by patients (Lupton, 2016). Our findings demonstrate that digital self-monitoring and the measurable and quantifiable data it provides, will not automatically add value to the daily lives of patients. In our study, the digital self-monitoring appeared to fit some patients’ lifeworld, whereas others did not perceive self-monitoring as useful or meaningful due to their established knowledge, practices and routines for managing their MS.

By studying patients’ use of digital self-monitoring in the context of a scientific study and how patients’ engagement with these technologies was shaped by this context, we point out some critical considerations for the design of scientific studies that evaluate digital self-monitoring technologies. First, adherence rates found in a scientifically controlled context do not necessarily resemble adherence rates in real-life practices. The patients we interviewed felt a responsibility to contribute to scientific research and were therefore motivated to adhere to the technologies and to obey the self-monitoring instructions (cf. Presset et al., 2021). However, they thought they would be less adherent if they were not participating in a scientific study. Digital self-monitoring technologies are known to be frequently abandoned by their users (Lazar et al., 2015; Shin et al., 2019), and a high level of adherence to these technologies in the context of a scientific study does not guarantee their long-term use when the motivation to contribute to research is absent.

Second, if we extrapolate from our study, the suitability of conventional scientific and clinical study designs, such as randomized controlled trials, for the evaluation of technologies that patients use at home can be questioned (Pols, 2012). Until the time of writing, many scientific studies, including the APPS MS study, have required a one-size-fits-all approach, for example, standardized research protocols in which patients’ technology use is controlled to allow for quantitative comparisons and statistical analyses. However, these established research designs are not necessarily sensitive to the ambiguities and contingencies that are characteristic of patients’ lifeworld (Pols, 2012; Wieringa et al., 2018). As Pols (2012) has already argued, controlled and standardized study protocols do not provide space for the creative processes that patients engage in when building a relationship with a new technology, such as challenging, questioning or resisting what is being prescribed for them. Although it is important when evaluating medical innovations to investigate whether technologies are scientifically valid and reliable, patients’ willingness and ability to use these technologies as part of their everyday lives should also be assessed, which might require a different research design.

Third, we reiterate the challenge that is often discussed in the relevant sociological literature: how can patients’ experiential knowledge be integrated into scientific practices concerning the evaluation of medical innovations such as digital self-monitoring? On the one hand, ongoing initiatives and discussions are being conducted by funding agencies, policymakers and patient organizations, among others, that focus on opening up research and development trajectories to include the needs, wishes and knowledge of patients in decision-making processes (Boaz et al., 2016; Boenink et al., 2018; Greenhalgh et al., 2019). On the other hand, the quantitative and statistical evidence provided by standardized research designs remains the dominant type of knowledge on which stakeholders such as health insurance companies and healthcare providers still rely in their assessment of medical innovations (Wendrich and Krabbenborg, 2021). And researchers and technology developers may consider patients’ experiential knowledge to be a less legitimate and formalized form of knowledge than scientifically produced knowledge (Ledderer et al., 2019; Pols, 2012; Wieringa et al., 2018). Integrating patients’ knowledge into scientific practices might thus be hampered by established routines and historically grown “best practices” that are not easily changed because they are widely accepted (cf. Geels, 2020), such as dominant beliefs about what counts as evidence when new technologies are assessed (Wieringa et al., 2018).

By conducting our study, we have gained a better understanding of how patients engaged with digital self-monitoring technologies in their everyday life as part of a scientific study and how well these technologies fitted their lifeworld. This is a much-needed research effort, as there is a scarcity of empirical research on how patients with a chronic disease negotiate digital self-monitoring. Although we have acquired valuable insights, it is important to realize that we only conducted interviews at one moment in time. Moreover, we inquired into patients’ experiences retrospectively. As the respondents had to recall their experiences 6 months on average after their participation in the scientific study, some of them found it difficult to remember their experiences accurately and fully. To gain a better understanding of the long-term dynamics of the use and non-use of digital self-monitoring, we recommend that researchers set up a longitudinal study in which multiple moments after the introduction of the technology in patients’ daily practice are studied. Providing patients with a diary could be helpful in such an approach, as taking notes makes it easier and more tangible for respondents to trace their memories during an interview (Janssens et al., 2018). Another consideration is that we studied a limited sample of patients with a chronic disease, namely MS patients who were willing to participate in a scientific study. As different groups of patients have a different lifeworld, we cannot make generalizing statements based on our sample. Future research should therefore also study other patient groups and other contexts of technology use. To conclude, empirical inquiry into the use of digital self-monitoring by patients with a chronic disease, which we did in this paper, can uncover similarities and discrepancies between patients’ lived experiences and developers’ assumptions and expectations.
